# Renal Tumor Necrosis Factor α Contributes to Hypertension in Dahl Salt-Sensitive Rats

**DOI:** 10.1038/srep21960

**Published:** 2016-02-26

**Authors:** Baorui Huang, Yuan Cheng, Kristie Usa, Yong Liu, Maria Angeles Baker, David L. Mattson, Yongcheng He, Niansong Wang, Mingyu Liang

**Affiliations:** 1 Department of Nephrology and Rheumatology, Shanghai Jiaotong University Affiliated Sixth People’s Hospital, Shanghai, P.R.China; 2 Medical College of Soochow University, Suzhou, Jiangsu, P.R.China; 3 Center of Systems Molecular Medicine, Milwaukee, WI, USA; 4 Department of Physiology, Medical College of Wisconsin, Milwaukee, WI, USA; 5 Department of Nephrology, Shenzhen Second People’s Hospital and the First Affiliated Hospital of Shenzhen University, Shenzhen, China

## Abstract

Tumor necrosis factor α (TNFα) is a major proinflammatory cytokine and its level is elevated in hypertensive states. Inflammation occurs in the kidneys during the development of hypertension. We hypothesized that TNFα specifically in the kidney contributes to the development of hypertension and renal injury in Dahl salt-sensitive (SS) rats, a widely used model of human salt-sensitive hypertension and renal injury. SS rats were chronically instrumented for renal interstitial infusion and blood pressure measurement in conscious, freely moving state. Gene expression was measured using real-time PCR and renal injury assessed with histological analysis. The abundance of TNFα in the renal medulla of SS rats, but not the salt-insensitive congenic SS.13^BN26^ rats, was significantly increased when rats had been fed a high-salt diet for 7 days (n = 6 or 9, p < 0.01). The abundance of TNFα receptors in the renal medulla was significantly higher in SS rats than SS.13^BN26^ rats. Renal interstitial administration of Etanercept, an inhibitor of TNFα, significantly attenuated the development of hypertension in SS rats on a high-salt diet (n = 7–8, p < 0.05). Glomerulosclerosis and interstitial fibrosis were also significantly ameliorated. These findings indicate intrarenal TNFα contributes to the development of hypertension and renal injury in SS rats.

Hypertension is a major public health issue^1^. Salt is one of the major environmental factors that contribute to the development of hypertension^2^. The Dahl salt-sensitive (SS) rat is the most commonly used polygenic animal model of human salt-sensitive hypertension and renal injury^3^.

Inflammation has been proposed as an important contributor to the development of hypertension, including salt-induced hypertension in SS rats. Circulating leukocyte counts were elevated significantly in SS rats fed a high-salt diet compared with Dahl salt-resistant rats^4^. Renal interstitial inflammation and infiltration of immune cells are prominent in SS rats treated with high-salt diets^5^,^6^. Pharmacological or genetic manipulations that inhibit inflammation and immune response reduce renal interstitial infiltration of immune cells and attenuate salt-induced hypertension and renal injury in SS rats as well as in other models^7^,^8^,^9^,^10^.

Tumor necrosis factor α (TNFα) is a major proinflammatory cytokine that has been reported to be elevated in hypertensive patients^11^,^12^. Systemic administration of Etanercept, a soluble recombinant fusion protein that blocks the functional effect of TNFα, attenuates the development of hypertension and renal injury in several models^13^,^14^,^15^.

It is not clear, however, whether TNFα specifically in the kidney contributes to hypertension. In the present study, we analyzed the expression levels of TNFα and its receptors in the kidneys of SS rats. We then examined the contribution of intrarenal TNFα to hypertension by infusing Etanercept directly and locally into the renal interstitium. Renal interstitial infusion or injection is a well-established experimental technique for administering agents specifically into the kidney^16^,^17^,^18^,^19^. The technique requires sophisticated chronic instrumentation when it is applied in conscious animals.

## Methods

### Animals

The animal experiments were performed in accordance with the recommendations in the Guide for the Care and Use of Laboratory Animals of the National Institutes of Health. The animal protocols were approved by the Institutional Animal Care and Use Committee at the Medical College of Wisconsin (AUA206). Inbred SS rats and congenic SS.BN-(D13Rat151-D13Rat197)/Mcwi (also called SS.13^BN26^) rats with reduced blood pressure salt-sensitivity were produced at the Medical College of Wisconsin. Rats were on a 12:12 h light-dark cycle and had unlimited access to water. Male SS and SS.13^BN26^ rats were maintained on the AIN-76A diet containing 0.4% NaCl as described previously^20^,^21^,^22^,^23^,^24^,^25^.

### Real-time PCR

Kidney tissues were collected, RNA extracted, and real-time PCR performed as described previously^22^,^26^,^27^,^28^. 18S rRNA was used as internal normalizer. Primer sequences are shown in Table 1.

### Chronic instrumentation for renal interstitial infusion and blood pressure measurement

Chronic instrumentation of rats for renal interstitial infusion and blood pressure measurement was performed as we described previously^24^,^28^,^29^,^30^,^31^. Briefly, 6 weeks old SS rats underwent uninephrectomy to remove the right kidney, allowing the remaining kidney to be the sole determinant of renal function of the rat. Following one week of recovery, an infusion catheter was implanted into the interstitium of the remaining left kidney and another catheter was implanted into the left femoral artery. Both catheters were exteriorized from the back of the neck and connected to an infusion pump or a blood pressure transducer using a swivel. This preparation allows chronic renal interstitial infusion and arterial blood pressure measurement in conscious, freely moving rats. Rats were allowed another week to recover before the initiation of daily blood pressure recording through the arterial catheter. After a stable baseline of blood pressure was obtained for 3 days, continuous renal interstitial infusion of TNFα inhibitor Etanercept (Enbrel, Amgen, Thousand Oaks, CA) at a dose of 0.25 mg/kg/day, or saline control, was initiated. After another 3 days, rats were switched from the 0.4% NaCl diet to a 4% NaCl diet (Dyets). Interstitial infusion and blood pressure measurement continued for another 8 days before the rats were euthanized and tissues collected.

### Histological analysis

The kidneys were fixed in 10% phosphate-buffered formalin for 24 hours at room temperature, and then embedded in paraffin. Sections were prepared with 3 μm thickness and stained with Masson’s trichrome staining. All the slides were scanned by Nanozoomer digital pathology and were analyzed by Metamorph offline version 7.7.0.0 software. Glomeruli (90 to 120 per slide) were evaluated (scored from 0 to 4) on the basis of the degree of glomerulosclerosis as previously described^32^. Interstitial fibrosis, tubular casts, and urinary protein and albumin excretion was analyzed as we described previously^33^,^34^,^35^.

### Immunohistochemistry

Immunohistochemistry was performed in formalin-fixed, paraffin-embedded kidney sections from SS rats (n = 6–9), following procedures similar to what we described previously^33^,^34^,^35^. The primary antibodies, all from Abcam, and the dilution used were ab6671 for TNFα, 1:100, ab19139 for TNF receptor I, 1:1500, and ab109322 for TNF receptor II, 1:100.

### Statistics

Student t-test or 2-way RM ANOVA was used to analyze data. P < 0.05 was considered significant. Data are shown as mean ± SEM.

## Results

**Figure 1:**
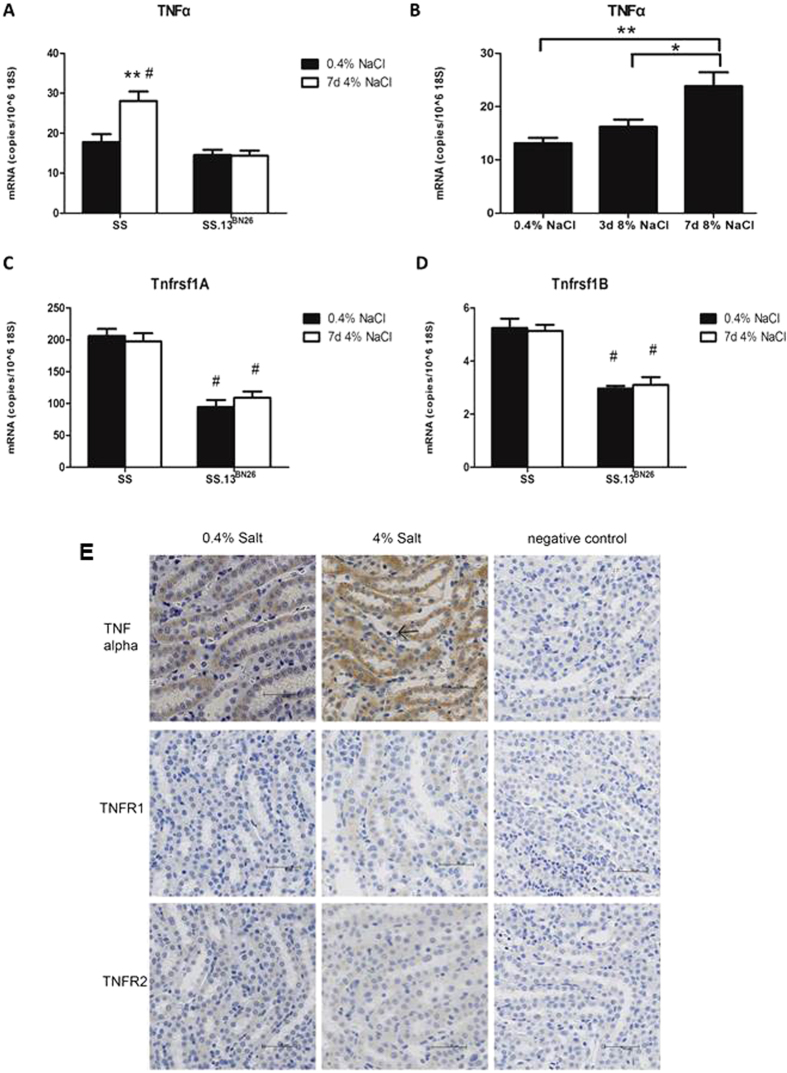
Abundance of TNFα and its receptors Tnfrsf1A and Tnfrsf1B in the renal medulla of SS and SS.13^BN26^ rats. (**A**) TNFα mRNA abundance in SS rats increased significantly when treated with a 4% NaCl diet for 7 days compared with SS rats on a 0.4% NaCl diet (n = 9, **P < 0.01) or salt-insensitive SS.13^BN26^ rats (#P < 0.01). (**B**) TNFα abundance in SS rats increased significantly after 8% NaCl diet for 7 days (n = 6, **P < 0.01 vs. 0.4% NaCl diet; *P < 0.05 vs. 3 days of 8% NaCl diet). (**C**,**D**) Abundance of TNFα receptors Tnfrsf1A and Tnfrsf1B were significantly higher in SS rats than in SS.13^BN26^ rats (n = 9, #P < 0.01 vs. SS). (**E**) Immunohistochemistry analysis. Representative images of renal medulla regions from SS rats maintained on the 0.4% NaCl diet or fed the 4% NaCl diet for 7 days (n = 6–9) are shown. The primary antibody was omitted in negative control. Brown color indicates positive staining. The arrow points to cells that appear to be infiltrating inflammatory cells. Scale bar is 50 μm.

The expression of TNFα and its receptors in the renal medulla of SS rats were examined using real-time PCR. Congenic SS.13^BN26^ rats, which we have shown to exhibit a lower level of blood pressure salt-sensitivity compared to SS rats^25^,^36^, were used as salt-insensitive control. TNFα abundance increased significantly in SS rats treated with 4% (n = 9) or 8% (n = 6) NaCl diet for 7 days, and this change occurred between 3 and 7 days of 8% NaCl diet (Fig. 1A,B). TNFα abundance was not altered by 7 days of 4% NaCl diet in SS.13^BN26^ rats (Fig. 1A). The mRNA abundance of TNFα receptors Tnfrsf1A and Tnfrsf1B was significantly higher in SS rats than in SS.13^BN26^ rats (Fig. 1C,D). However, treatment with the 4% NaCl diet for 7 days did not significantly alter the mRNA abundance of Tnfrsf1A or Tnfrsf1B in either rat strain. Immunohistochemistry analysis in the renal medulla of SS rats indicated the presence of TNFα protein in tubular cells and possibly infiltrating inflammatory cells, particularly after the high-salt diet treatment (Fig. 1E). TNF receptor II, encoded by Tnfrsf1B, was detectable in tubular cells in the renal medulla, while TNF receptor I, encoded by Tnfrsf1A, was detectable only in tubules in rats after the high-salt diet treatment.

**Figure 2:**
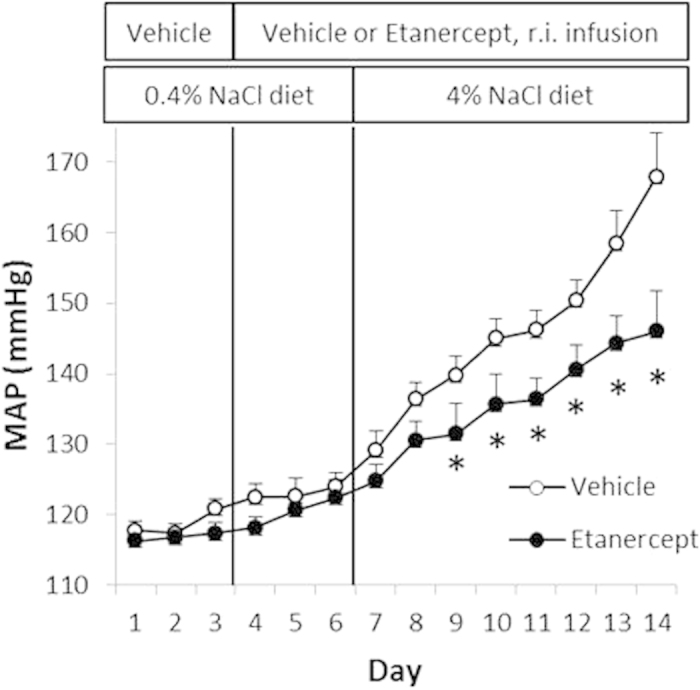
Renal interstitial administration of Etanercept attenuated the development of salt-induced hypertension in SS rats. Uninephrectomized SS rats received continuous renal interstitial infusion of saline vehicle or Etanercept (0.25 mg/kg/day) and were fed a 0.4% or 4% NaCl diet for the indicated durations. Mean arterial blood pressure (MAP) was measured in conscious, freely moving rats using indwelling femoral arterial catheter. N = 7–8, *p < 0.05 vs. vehicle-treated rats.

We examined if intrarenal TNFα contributed to hypertension in SS rats by treating uninephrectomized SS rats with renal interstitial infusion of the TNFα inhibitor Etanercept. Uninephrectomy made the remaining kidney receiving Etanercept the sole determinant of whole body renal function. Renal interstitial administration of Etanercept for 3 days while the rats were maintained on the 0.4% NaCl diet did not have a significant effect on mean arterial pressure. However, Etanercept significantly attenuated hypertension after the rats were switched to the 4% NaCl diet (n = 7–8, P < 0.05 vs. saline control on days 3 to 8 on the 4% NaCl diet, two-way RM ANOVA followed by Holm-Sidak test) (Fig. 2). The effect of Etanercept reached 22 mmHg at 8 days after the rats were switched to the high-salt diet.

**Figure 3:**
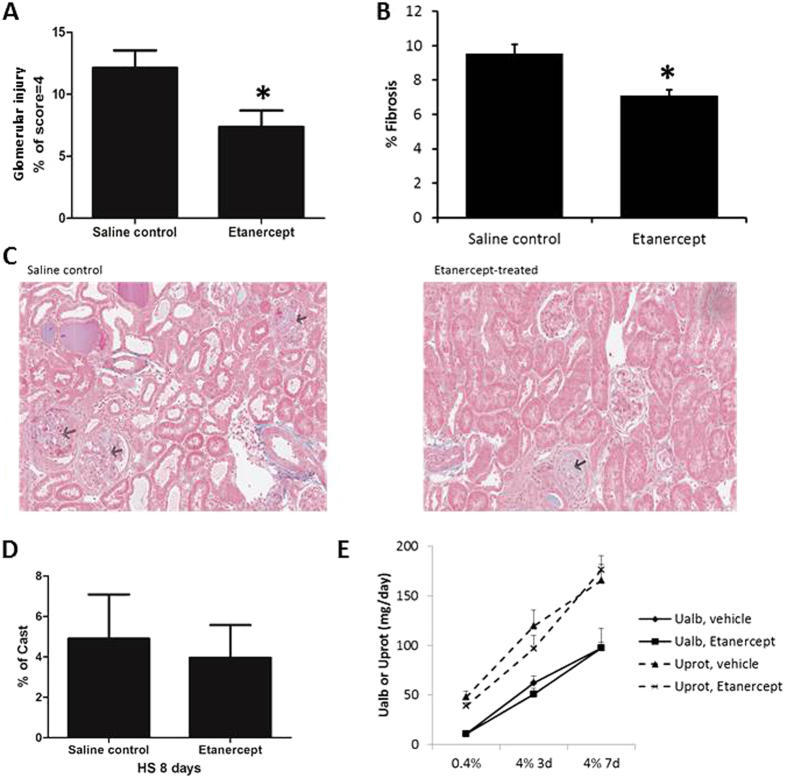
Renal interstitial administration of Etanerceptin SS rats ameliorated glomerulosclerosis and renal interstitial fibrosis. (**A**) Percent of glomeruli that were severely damaged (scored 4). (**B**) Percent of renal cortical area that was fibrotic. (**C**) Representative images of kidney sections. Arrows point to injured glomeruli. (**D**) Percent of kidney section area occupied by tubular casts. E. Urinary 24 hour excretion of albumin (Ualb) or total protein (Uprot). N = 4–8, *P < 0.05 vs. vehicle-treated rats.

SS rats develop substantial renal injury in addition to hypertension, a characteristic that resembles hypertensive African Americans^28^. Renal interstitial administration of Etanercept significantly reduced the percentage of glomeruli that were severely damaged and the percent area of the renal cortex that were fibrotic (Fig. 3A–C). The Etanercept treatment did not significantly change the percent area of kidney sections occupied by tubular casts or 24 hour urinary excretion of albumin or protein (Fig. 3D,E).

## Discussion

The major new finding of the present study was that administration of Etanercept directly into the renal interstitium significantly attenuated salt-induced hypertension in SS rats. The anti-hypertensive effect of Etanercept that we observed is likely the consequence of TNFα inhibition specifically in the kidney, rather than any systemic inhibition of TNFα by any Etanercept spilled over into the systemic circulation. Experiments using radiolabeled compounds have demonstrated that compounds infused into the renal interstitium largely stayed in the infused kidney^37^. Even if a small fraction of Etanercept did spill over into the systemic circulation, the spill-over is unlikely to account for the blood pressure effect we observed. The doses of Etanercept used for systemic administration are typically 5 to 10 times higher than the entire dose infused into the renal interstitium in the present study^13^,^14^,^38^,^39^. Note though the renal interstitial administration was used primarily to address mechanistic questions regarding the contribution of the kidney, not as a therapeutic approach.

Prior to the present study, it was not clear whether TNFα specifically in the kidney played a significant role in the development of hypertension. Several studies have reported that systemic treatment with TNFα inhibitor Etanercept altered the progression of hypertension^13^,^40^. However, it was not clear whether it was systemic or local TNFα that was involved. Moreover, whether TNFα contributes to increasing or, in some cases, decreasing blood pressure appears to depend on the level of TNFα that is present^41^. The findings of the current study suggest it would be important to consider levels of TNFα in the kidney locally. TNFα is unlikely to be the only proinflammatory cytokine involved. It is important to consider the role of other proinflammatory factors.

Previous studies have reported an increase of TNFα in the kidneys of SS rats after 5 weeks of 4% NaCl diet^42^. Our data indicated that up-regulation of renal TNFα in SS rats occurred early within 7 days of exposure to a high-salt diet. TNFα in the kidney could come from several sources including macrophages and lymphocytes that have infiltrated the kidney, resident cells in the kidney, and circulating TNFα. TNFα produced by resident epithelial cells in the kidney may play a physiological role in regulating electrolyte homeostasis^43^. In the present study, TNFα was detected in tubular cells and possibly infiltrating inflammatory cells in the renal medulla of SS rats particularly after the high-salt diet treatment, although relative contribution of each cell type remains to be determined. We do not know whether or how much TNFα released from the renal medulla contributes to the increase in systemic TNFα. The downstream mechanism by which renal TNFα contributes to hypertension in SS rats also remains to be investigated.

## Additional Information

**How to cite this article**: Huang, B. *et al.* Renal Tumor Necrosis Factor α Contributes to Hypertension in Dahl Salt-Sensitive Rats. *Sci. Rep.* **6**, 21960; doi: 10.1038/srep21960 (2016).

## Figures and Tables

**Table 1:** Real-time PCR primer sequences.

Gene name		Primer sequence
18s rRNA	forward primer	5′-CGGCTACCACATCCAAGGAA- 3′
	reverse primer	5′-CCTGTATTGTTATTTTTCGTCACTACCT- 3′
TNFα	forward primer	5′-TGGGCTCCCTCTCATCAGTTC- 3′
	reverse primer	5′-TCCGCTTGGTGGTTTGCTAC- 3′
Tnfrsf1A	forward primer	5′-CAAAGAGGTGGAGGGTGAAGG- 3′
	reverse primer	5′-ACTGAAGCGTGGGGTGGTG- 3′
Tnfrsf1B	forward primer	5′-TTAGAGGAGCCCAGTTGTTACCA- 3′
	reverse primer	5′-GACGTGTCCCTTCGCTTCTC- 3′
